# Nurses’ rationale for underreporting of patient and visitor perpetrated workplace violence: a systematic review

**DOI:** 10.1186/s12912-023-01226-8

**Published:** 2023-04-23

**Authors:** Cara Spencer, Jamie Sitarz, June Fouse, Kristen DeSanto

**Affiliations:** 1 UCHealth, Aurora, CO USA; 2 UCHealth Cancer Center, Highlands Ranch, CO USA; 3 University of Colorado Hospital, Aurora, CO USA; 4 University of Colorado Anschutz Medical Campus, Strauss Health Sciences Library, Aurora, CO USA

**Keywords:** Underreporting, Reporting, Workplace Violence, Nursing, Patient Perpetrated, Visitor Perpetrated, Aggression

## Abstract

**Background:**

Patient and visitor perpetrated workplace violence (WPV) is a problem within healthcare and is known to be underreported by nurses and other healthcare workers. However, there are multiple and diverse reasons identified in the literature as to why nurses do not report. This systematic review aimed to investigate nurses’ reasons and rationale related to underreporting of violence that occurs in the workplace.

**Methods:**

Following PRISMA guidelines for systematic review reporting, studies conducted between 2011 and early 2022 were identified from MEDLINE, CINAHL, APA PsychInfo, and Psychological and Behavioral Sciences Collection via EBSCOHost. Quantitative studies related to patient and visitor perpetrated violence containing explanations, reasons, or rationale related to underreporting were included.

**Results:**

After quality appraisals, 19 studies representing 16 countries were included. The resulting categories identified nursing, management, and organizational factors. The most prominent nursing factors included nurses’ fear of consequences after reporting, nurses’ perceptions, and their lack of knowledge about the reporting process. Common management factors which contributed to nursing underreporting included lack of visible changes after reporting, non-supportive culture in which to report, and the lack of penalties for perpetrators. Organizational factors included the lack of policies/procedures/training for WPV, as well as a lack of an efficient and user-friendly reporting system. Supportive interventions from management, organizations, and community sources were summarized to provide insight to improve nurse reporting of WPV events.

**Conclusion:**

Underreporting of WPV is a complex and multi-faceted problem. An investigation into the rationale for underreporting a workplace violent event illustrates nurses, management, and organizations contribute to the problem. Clear and actionable interventions such as educational support for staff and the development of a clear and concise reporting processes are recommended to encourage staff reporting and to help address WPV in healthcare.

## Background

Workplace violence is a pervasive global problem within healthcare [1, 2]. As defined by the United States Occupational Safety and Health Administration [3], WPV is “any act or threat of physical violence, harassment, intimidation, or other threatening disruptive behavior that occurs at the worksite … and includes threats, verbal abuse, physical assaults, and even homicide…” [3]. Patients and visitors are the main perpetrators of WPV in healthcare regardless if the event was verbal, psychological, physical, or sexual in nature [4].

Nurses are at the highest risk of WPV due to their direct contact with patients and visitors compared to other professional roles in healthcare [5]. One out of every five nurses experiences a physically violent event each year [6]. Considering the World Health Organization (WHO) estimates there are 27.9 million nurses across the globe [7], this equates to more than five and a half million nurses experiencing a physically violent event while providing patient care each year.

While the number of events is staggering, it may not fully encompass the magnitude of the problem due to underreporting. Underreporting is defined as a failure of the victimized employee to report events to employers, police, or other officials [8]. OSHA estimates that two-thirds of all workplace-related injuries and illnesses go unreported [9]. And Hamed and Konstantinidis [10] found underreporting is a pervasive challenge for nurses and poses a significant safety issue for both patients and staff.

More specific to workplace violence against nurses, Kvas et al. [11] found only 6.5% and Arnetz et al. [12] found only 12% of events were formally reported while other sources state reporting of WPV events was as low as 3% [13]. More recently, as healthcare has gained understanding about WPV, Garg et al. [14] found 23.5% of events were reported and the American Nurses Association (ANA) states 20 to 60% of events are reported [15]. This variability of underreporting may be related to the concept of underreporting itself. Not only is WPV underreported, but it is possible that underreporting is also underreported.

The Joint Commission (TJC) and the ANA support reporting of events as this step is critical to address WPV [16]. Any violence that is verbal, nonverbal, written, or physical is to be reported [16]. When an organization can track violent events, the ability to address WPV is improved [15, 16].

Moreover, different forms of WPV are not reported equally or proportionately. Physically violent events are more likely to be reported, especially if there was an injury or lost time from work [17]. However, Schablon et al. [18] found that each year, 94% of nurses experience verbal aggression. As often as verbal violence occurs, it is likely to be underreported [19]. Verbal violence, “near-misses,” and other events perceived to not be sufficiently serious enough to warrant a report, are most frequently underreported [19, 20].

Underreporting is considered a significant barrier to addressing WPV. Simply stated, “what goes unreported, goes unfixed” [1]. Not only does underreporting underestimate the magnitude of the problem, but prevention and interventions may address only what is known. Without a full understanding of WPV, efforts and interventions may miss the full spectrum of the problem [1].

## Objective

A comprehensive review of recent literature to understand the causes of underreporting of WPV events may assist healthcare leaders to address the problem. As a multi-faceted and global concern for nursing, WPV in healthcare needs clear identification of nurses’ perspectives of underreporting of patient and visitor perpetrated violence to ensure mitigating interventions can be initiated. Therefore, the purpose of this systematic review was to investigate and summarize recent literature regarding nurses’ reasons or rationale for underreporting patient or visitor perpetrated WPV in healthcare. Once identified, an analysis of the selected studies, including interventions and recommendations, may shed light on the phenomenon. Therefore, the research question developed by the authors was as follows:

- Why do nurses underreport patient or visitor perpetrated WPV?

## Method

### Study design

The research question indicated a systematic review was appropriate to address the reasons and rationale for underreporting by nurses after WPV events. The systematic review was designed and based upon the international guideline of The Preferred Reporting Items for Systematic Reviews and Meta-Analyses (PRISMA) [21].

From the data, categories and sub-categories were necessary to organize the data. Content analysis illuminated three categories contributory to underreporting: nursing, management, and the organization. Sub-categories were identified for each factor; eight for nursing, three for leadership, and four for the organization.

### Eligibility criteria

Study eligibility was based on current, global perspectives of nurses in the past 10 years, to include an adequate sample of studies before the COVID pandemic, as not to skew the results of the review. Inclusion criteria incorporated studies written in English, peer-reviewed articles published between January 1, 2011, and March 24, 2022, and studies with clearly defined designs and methods. The included studies focused on patient or visitor perpetrated violence. The subjects or participants needed to be inclusive of nurses, providing reasons or rationale for the underreporting of WPV in a healthcare setting. Studies were excluded if sexually violent events were the primary focus of the project, if the subjects or investigators were students, or if the study only presented prevalence rates of underreporting without additional information related to underreporting rationale. Additionally, qualitative studies, editorials, commentaries, and conference abstracts were excluded as authors determined there were substantial qualitative studies to merit a separate analysis.

### Search strategy and data sources

**Fig. 1:**
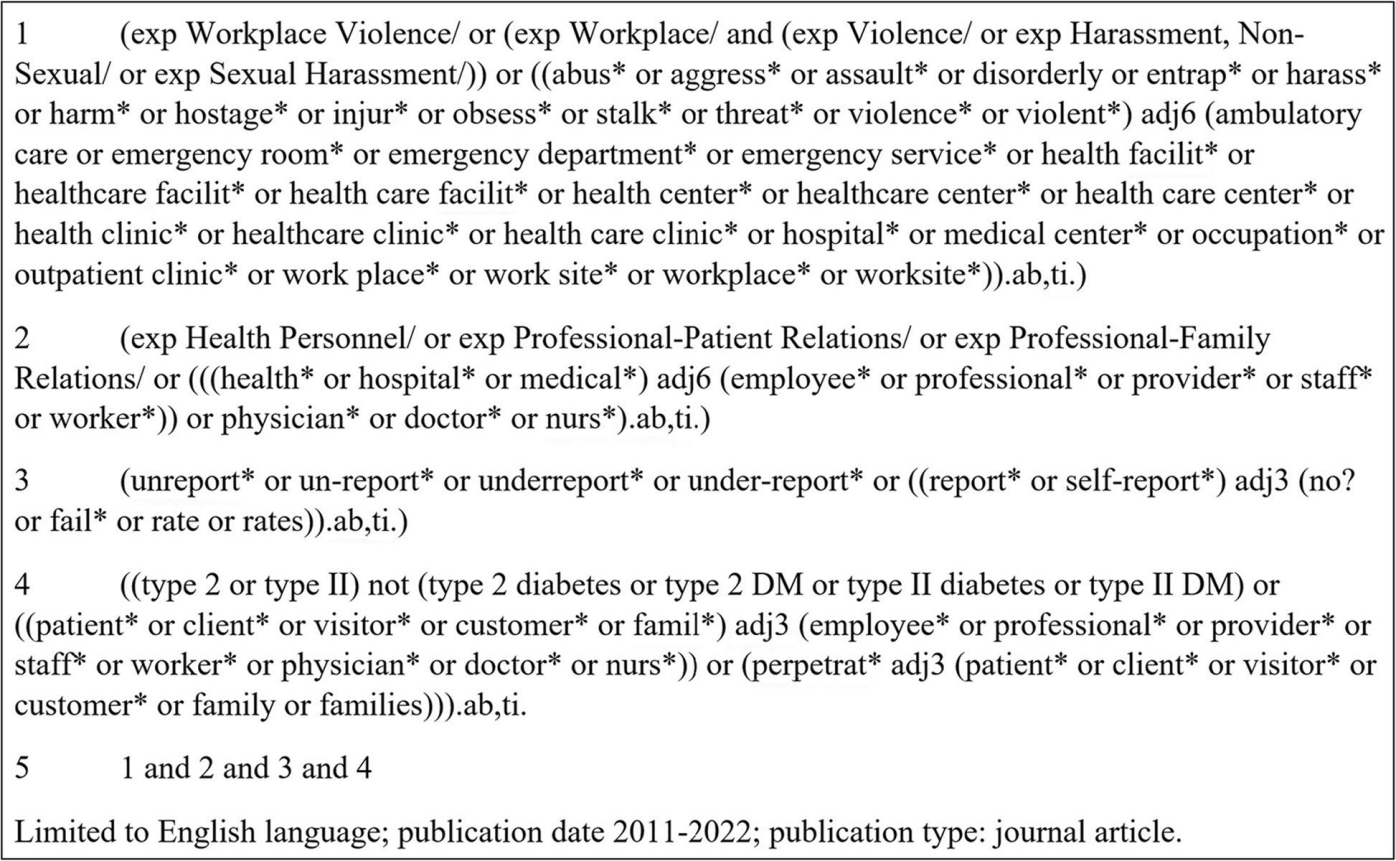
Ovid MEDLINE® search strategy for patient or visitor perpetrated WPV

A comprehensive literature search was performed by a medical librarian on September 3, 2021, and updated again on March 24, 2022, for additional recent studies. Databases were searched using a combination of keywords and database-specific indexing terms (when applicable) representing the concepts of type II workplace violence, i.e., patient and visitor perpetrated WPV, healthcare personnel, and underreporting. See Fig. 1 for the Ovid MEDLINE® search strategy.

**Fig. 2:**
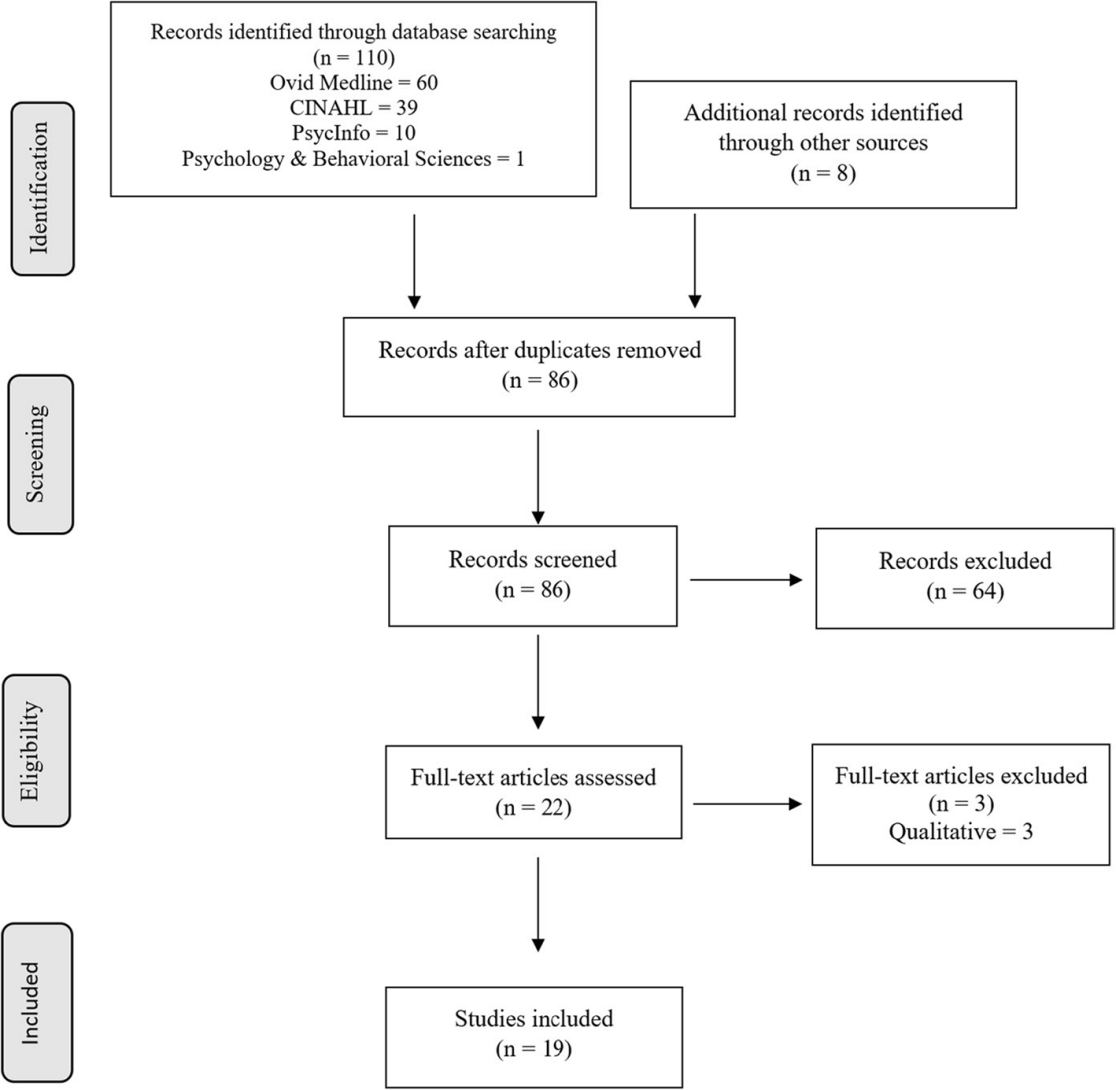
PRISMA Flow Diagram of the literature search process

The following electronic databases were searched for eligible studies: MEDLINE (via Ovid MEDLINE® ALL), CINAHL (Cumulative Index to Nursing and Allied Health Literature, via EBSCOhost), APA PsycInfo (via Ovid), and Psychology and Behavioral Sciences Collection (via EBSCOHost). Once an initial set of articles was identified for inclusion in the review (Algwaiz et al. [22]; Alsharari et al. [23]; Babiarczyk et al. [24]; Fisekovic Kremic et al. [25]; Kitaneh et al. [26]; Li et al. [6]; Zahra et al. [2]; Pompeii et al. [27]; Sato et al. [28]; Song et al. [17]), the librarian conducted a search for those ten articles in Google Scholar (https://scholar.google.com/) on March 24, 2022, examining the “cited by” lists to identify additional relevant articles. All database search results were exported to and de-duplicated in EndNote™ 20 (Clarivate Analytics, Philadelphia, PA). After removing duplicates, 86 articles remained, 64 of which were excluded based on title and abstract. Twenty-two articles were assessed in full text and three were excluded due to qualitative study methodology, leaving 19 studies included in the analysis. See Fig. 2 for the PRISMA Flow Diagram of the literature searching processes.

### Quality appraisal

Quality appraisals of the selected literature were conducted by two authors using the Scottish Intercollegiate Guidelines Network (SIGN) checklists [29]. SIGN utilizes different checklists applicable to the methodology used in the study being assessed. Thus, the appropriate checklist was used based on each study appraised. These quality appraisal tools evaluated the study design to assess for significant risk of bias in the results or conclusions drawn within the study. Scoring of each study used “high quality, acceptable, or unacceptable.” Any discordance between the first two assessments was resolved by a third author.

The data extraction table included: author, year of publication, country, study design, number of participants, data collection method, and quality assessment. Three authors independently extracted the data from each study, summarized the data on an Excel spreadsheet, and then shared their results. In the case of disagreement, the authors discussed the data until an agreement was found.

The authors chose content analysis to analyze the data to determine the presence of certain concepts, themes, or categories. After reading and interpreting the content, the data was categorized according to their similarities and differences and categories and sub-categories were identified. One author identified the categories and sub-categories. These were discussed and agreed upon by the other three authors during peer review.

## Results

### Description of included studies

Of the 19 included studies, 17 countries were represented: China (*n* = 2), India (*n* = 1), Indonesia (*n* = 1), Italy (*n* = 1), Japan (*n* = 1), Jordan (*n* = 2), Nigeria (*n* = 1), Palestine (*n* = 1), Saudi Arabia (*n* = 3), Serbia (*n* = 1), Slovenia (*n* = 1), United States (*n* = 3), and multinational (*n* = 1). The multinational studies included Poland, Czech Republic, Slovak Republic, Turkey, and Spain. Most were cross-sectional survey studies (79%), and nursing was represented in the collective or identified as the independent profession being studied. Quality assessments revealed that 16 studies scored as high quality and three as acceptable. None of the studies were eliminated due to unacceptable scoring. See Table 1 for a summary of study characteristics. Incidental findings not related to the research question are summarized in Table 3 in Discussion.

**Table 1:** Summary of study characteristics included

**Study (year)**	**Country**	**Design of study**	**Participants (n)**	**Method of data collection**	**Quality assessment**
Zahra AN et al. (2018) [2]	Indonesia	Quantitative	RN (169)	Survey	High Quality
Arnetz JE et al. (2015) [12]	USA	Quantitative	Employees and RN’s (446)	Survey and retrospective reports	Acceptable
Copeland D et al. (2017) [13]	USA	Quantitative	ED staff and RN’s (147)	Survey	High Quality
Song C et al. (2020) [17]	China	Quantitative	RN (266)	Survey	High Quality
Al-Azzam M et al. (2018) [30]	Jordan	Quantitative	RN (137)	Questionnaire	High Quality
AlBashtawy M et al. (20150 [31]	Jordan	Quantitative	RN (227)	Survey	High Quality
Algwaiz WM et al. (2012) [22]	Saudi Arabia	Quantitative	Physician and RN’s (383)	Survey	High Quality
Alsharari AF et al. (2021) [23]	Saudi Arabia	Quantitative	RN (849)	Survey	High Quality
Alsmael MM et al. (2020) [32]	Saudi Arabia	Quantitative	HCW and RN’s (360)	Survey	High Quality
Babiarczyk B et al. (2020) [24]	Poland, Spain, Czech Republic, Slovak Republic, Turkey	Quantitative	RN (1089)	Survey	High Quality
Cannavò M et al. (2019) [33]	Italy	Quantitative	HCW and RN’s (322)	Survey	High Quality
Douglas K et al. (2019) [34]	Nigeria	Quantitative	RN (200)	Questionnaire	Acceptable
Fisekovic Kremic MB et al. (2016) [25]	Serbia	Quantitative	Staff and RN’s (1526)	Survey	High Quality
Garg R et al. (2019) [14]	India	Quantitative	Physicians, RNs, HCWs (394)	Survey	High Quality
Kitaneh M et al. (2012) [26]	Palestine	Quantitative	Physician and RN’s (240)	Questionnaire	High Quality
Kvas A et al. (2014) [11]	Slovenia	Quantitative	RN (692)	Survey and Questionnaire	Acceptable
Li P et al. (2018) [6]	China	Quantitative	GP’s and RN’s (830)	Survey	High Quality
Pompeii LA et al. (2016) [27]	USA	Mixed method	HCW and RN’s (5385)	Survey and Focus Groups	High Quality
Sato K et al. (2013) [28]	Japan	Quantitative	RN (1385)	Survey	High Quality

*HCW* Healthcare Workers, *RN* Register Nurse, *ED* Emergency Department, *GP* General Practitioners

### Underreporting factors

The data revealed multiple factors associated with underreporting of WPV and were divided into nursing, management, and organizational categories. Each of these categories were separated into sub-categories; eight for nursing, three for management, and four for organizational. Results are outlined in Table 2. Authors included all factors that each study identified as contributing to underreporting of WPV to enhance a complete understanding of the subject.

**Table 2:** Factors: Rationale for underreporting of workplace violence

**Nursing Categories**	
Personal Characteristics [27, 28]	• Female
	• Younger in age
	• Newer nurse, less work experience
	• Working alone
	• Caring or empathetic personality
	• Working in a specialty care area
	• More violent experiences, the less likely to report
	• Desensitized to violent patients
	• Personal bias against reporting
Fear [6, 11–13, 17, 22–28, 31, 32, 34]	• Losing job
	• Serious consequences
	• Legal consequences
	• Poor job performance evaluation
	• Reprisals from management
	• Revenge or retaliation
	• Not believed
	• Blaming victim
	• Lack of support from colleagues
	• Low patient satisfaction scores
	• Causing harm to the patient by reporting
Lack of Knowledge About Reporting Workplace Violence [12–14, 17, 24, 25, 32, 33]	• Do not know how to report
	• What to report (ambiguous)
	• Who to report to
	• Never instructed on the reporting process/did not know there was a reporting process
	• Unsure what is considered “reporting” – Does documenting in the patient chart or verbally reporting to coworkers /supervisor count?
Time Constraints [12, 13, 17, 27, 34]	• Too time-consuming
	• Too busy
	• Inconvenient
	• Forget
The perception that Violence is Unavoidable [12, 13, 17, 22, 27, 28, 30, 31]	• “Part of the job”
	• Expected to “handle it”
	• Common to their patient care area
	• Not important
Perception of the Patient [17, 27, 28]	• Some patients cannot control their behavior
	• Violence not intentional
	• Patient apologized
Perception of the Level of Severity [6, 12, 13, 17, 22, 24, 27, 28, 31, 33]	• Physical violence is reported more often than verbal violence
	• Physical injury more likely to be reported
	• Verbal violence not considered violence or not severe enough to report
	• More likely to report if a weapon was used
	• Increased severity of an event, increased reporting
	• Mitigation of the violent incident is considered when deciding to report
Other Negative Perceptions [11, 13, 17, 24–26, 28, 31–33]	• Feelings of guilt or shame
	• Viewed as occurring due to the nurse’s actions
	• Pressure from colleagues not to report
	• Considered a bureaucratic task
	• Viewed as a preventable event
	• Previously reported and had a negative experience
**Management Categories**	
Unsatisfied with Outcome [11–13, 17, 22–28, 31–34]	• Lack of adequate response from management following a report
	• Causes or event itself not investigated
	• No positive changes because of reporting; useless
	• No consequences for the perpetrator
	• Dissatisfied with the resolution of events
Lack of Support to Report [2, 6, 13, 17, 26, 28, 32]	• No incentive to report
	• Discouraged from reporting
	• Not mandatory to report
	• Lack of manager support to report
Culture [13, 17, 28]	• Lack of willingness to defend nurses
	• Paying greater attention to patients rather than nursing staff
	• Not taken seriously
	• Reporting is not the norm; others do not report
**Organizational Categories**	
Lack of Policies and Procedures [26]	• Lack of clear and detailed policies and procedures that address workplace violence
Lack of a Reporting System [2, 6, 17, 26]	• Lack of a functioning and user-friendly reporting system for workplace violence
Lack of Training Programs [6, 26]	• Lack of mandatory organizational training on workplace violence
	• Lack of violence prevention training programs

## Discussion

This systematic review investigated quantitative studies which outlined reasons and rationale for nurses to underreport WPV events. As a global phenomenon, WPV underreporting is internationally pervasive. The 19 studies published between 2011 to early 2022 were homogenous in respect to methodology and surveyed nurses about their experiences with underreporting. Investigation into underreporting revealed three contributing factors/categories to underreporting: nurses, leaders, and organizations.

### Nurse factors that impact underreporting

Nurses’ characteristics may influence underreporting of WPV. Various studies suggest that younger female nurses, less experienced nurses, and nurses who experience a higher frequency of violence are less likely to report it [24, 32]. First, Sato et al. [28] found nurses with less experience may perceive themselves as less valued while developing their nursing competency and therefore feel their voice has less significance compared to their leaders. These characteristics mirror nurses who are most often targets of WPV. Although studies contain varying results, younger female nurses with less experience are most often the victims of WPV events [35]. Additionally, nurses who work alone, work in specialty areas, or are highly empathetic are also less likely to report WPV. As noted by Hanson et al. [36], home care nurses, who work alone, find themselves reliant upon their knowledge and skills to keep them safe against WPV. This independence and self-reliance may also translate into underreporting.

Most often, nurses underreport due to the perception that violence is “part of the job” which leads them to minimize the event [20, 22, 28, 37]. Although five of the 19 studies used the term “part of the job”, other studies describe this synonymously as “common to their care area” [13] or “de-sensitized to violent patients” [27]. Also, nurses were found to underreport if the event was not viewed as severe enough, the behavior was perceived as unintentional, no injury was incurred, or the perpetrator apologized. This is noted to be particularly true for verbal aggression, which has become so commonplace that it is accepted as part of the nature of the workplace [20]. These attitudes normalize violence, promote complacency towards WPV events, and appear to contribute to underreporting.

Emotions and feelings such as guilt and shame can also contribute to nursing underreporting and are noted in four studies. Emotions could affect reporting if the nurse perceived themselves, in any way, culpable for the event, anticipated the event, or feel they should have had knowledge or skills to combat or deescalate the situation [22]. Alas, these emotions may be misplaced as some violent episodes are unforeseeable and therefore unable to be thwarted [38].

Fear, a stronger emotion, was a significant reason for nurses to underreport as noted in 15 out of 19 studies. Fear can manifest from multiple sources. Not only can the nurse be fearful of perpetrator retaliation or revenge, but there can also be fear from colleagues or management. The nurse may fear a lack of collegial support or even judgment from peers, feel pressure to not report if peers do not, or fear being blamed for the event. Fears related to management include the fear of losing their job, managerial reprisal, poor job performance appraisals, or legal consequences if the nurse reports. The nurse may also be fearful of poor patient satisfaction scores or negative consequences from the perpetrator due to reporting the event.

Fear is a known issue with WPV and seems to parallel issues related to underreporting. Female nurses are more fearful of WPV compared to men [39]. This may be due to women typically being smaller in stature and lacking the large physical presence men possess to confront WPV events. Women are also noted to be psychologically more sensitive after events which can translate into compounding fear [39]. Additionally, nurses fear management reprisals. This may be related to leaders making decisions that reduce the negative impact on the hospital after a WPV event, but these decisions may be at the expense of nursing interests [32, 40].

Time constraints can also play a significant part in reporting WPV events. If nurses do not have the time to step away from patient care or perceive reporting as too time-consuming or inconvenient, reporting may be hindered. This is similar to underreporting of all errors or incidents within nursing. Hamed and Konstantinidis [10] found that lack of time, reporting processes that are too laborious, and work pressures present barriers to reporting incidents within nursing. Thus, the barrier of time to report events is not specific to WPV.

Other contributing aspects of WPV underreporting are in the realm of knowledge or lack of education. Nurses underreport when there is a lack of clear definition of what constitutes WPV, do not know who, where, or how to report an event, or if there is a cumbersome reporting system. This is similar to other incident reporting for nurses who state they have a lack of skills, knowledge, and training to file a report, and state they are uncertain as to what constitutes an error [10]. This lack of information may leave nurses vulnerable and uncertain as to the appropriate steps to take when an incident occurs, regardless of whether the event is related to WPV or not.

### Management factors that impact underreporting

Management is noted to be the most impactful factor in nurse underreporting of WPV events. As mentioned in 15 of the 19 studies, nurses underreport due to a lack of positive changes after filing a report and thus perceive reporting as “useless”. This may make victims hesitant to report again. However, Arnetz et al. [12] found nearly half of all healthcare workers provide only a verbal report to a colleague or supervisor. Although a staff member may believe the event was reported, formal documentation of the event may not have been provided to upper management who can initiate changes in policies, procedures, and endow resources to address WPV [12]. Thus, the staff member, who verbally reported the WPV event to their supervisor, is left with the perception that reporting is useless since no positive changes occurred. In essence, ineffective reporting by nurses leads to further underreporting.

Moreover, nurses underreport due to the lack of managerial investigation into the cause of the incident and dissatisfaction with the follow-through after a WPV event. This appears similar to other error/incident underreporting issues [10]. A noted difference between WPV events and other forms of incident reporting is the lack of consequences for the perpetrator of WPV. However, both error/incident reporting and WPV underreporting may stem from the nurse’s perception that they will be blamed for the event [10].

### Organization

For healthcare organizations, WPV is an evolving problem and requires infrastructure to support WPV safety and reporting. For organizations without established infrastructure such as WPV policies and procedures outlining reporting processes, efficient reporting systems, and resources to address WPV, underreporting persists unabated. Moreover, nurses underreport WPV events if the organization has not provided training on WPV resources and instruction on reporting processes in a supportive environment.

### Interventions

As a result of the systematic review on WPV underreporting, the authors discovered, summarized, and categorized potential WPV interventions into three categories: organizational, management, and community. Whereas the organization develops the infrastructure to combat WPV, such as with policies and procedures, management ensures these are conducted and creates a culture of non-judgmental approachability supporting the concept that WPV is never “part of the job”. Community leaders, such as those from professional organizations, law enforcement, government, and academic institutions, may join collaboratively beyond the confines of healthcare, to solve this complex and multidimensional problem.

Reporting and WPV prevention infrastructure have a significant mediating effect on the negative consequences after an event. Policies, procedures, reporting systems, and staff education are necessary to ensure pre-established processes provide protection for the staff and foster organizational trust [41]. Content associated with recommended interventions was an incidental finding and not part of the author’s research question, therefore not included in the results section of this systematic review. Many of the studies included in this review held recommendations to mitigate underreporting therefore that information was summarized for the reader’s benefit. Recommended interventions for healthcare leaders are summarized in Table 3.

**Table 3:** Summary of interventions to mitigate underreporting

**Organizational Interventions**	
Policy & Reporting System [2, 6, 11–13, 17, 22–28, 30, 32, 34]	• Consider a zero-tolerance WPV policy/campaign including signage regarding behavior expectations and consequences
	• Develop a clear and detailed WPV policy including definitions of WPV and formal reporting processes
	• Ensure the policy includes structure/process for immediate response and follow-up
	• Confirm that the reporting system is clear, effective, userfriendly, and not time-consuming
	• Evaluate the reporting system routinely for effectiveness and usability maintaining oversight by stakeholders
Education and Training [1, 2, 6, 11–13, 17, 22–25, 27, 28, 30–32, 34]	• Educate staff about the phenomenon of WPV in healthcare and its implications. Teach that everyone has the right to freedom from harm and that violence is not “just part of the job” • Inform about the magnitude of underreporting; how it minimizes the problem and puts nurses at risk in the future
	• Educate that reporting is essential for leadership to investigate incidents and for future allocation of resources
	• Ensure that institutional training is mandatory; included in the initial orientation and annual review and communicating that reporting is non-punitive and will not affect their annual job performance evaluations
	• Ensure training includes detailed WPV policy and reporting system
	• Provide prevention programs that teach situational awareness, risk factors, de-escalation techniques, teamwork training, therapeutic communication skills, conflict management skills, and collaborative care to decrease unmet patient needs
	• Provide training to managers on conflict resolution, early recognition of problems, and coaching skills
Staffing [2, 13, 22, 23, 30, 32]	• Ensure the presence and availability of security officers in high-risk areas
	• Maintain adequate healthcare & security staffing
Security Measures [13, 22, 23, 30]	• Perform risk assessments routinely to include workflow and overcrowding issues. Work to decrease wait times
	• Communicate behavior expectations to patients and family
	• Ensure that patients/families identified as high risk are communicated to all staff/departments
	• Enforce security and visitor policies
Collaboration [17, 22, 24, 27]	• Collaborate with leaders in other hospitals and institutions to share data, identify solutions, and implement improvements
	• Develop a WPV response team that will collaborate and review each report of WPV to provide a satisfactory response
	• Ensure all leaders are engaged
Support-Resources [23, 24, 26]	• Provide resources for staff affected by WPV; physical, emotional, psychological, and legal
	• Offer resources that help teach coping skills and decrease occupational stress
**Management Interventions**	
Create a Positive Unit Culture [17, 24, 32]	• Display a caring, engaged, supportive, approachable, and nonjudgmental attitude
	• Create an environment of valuing nurses’ input and a culture of preventing, recognizing, reporting, and addressing violence
	• Develop and enforce an open and blame-free culture around WPV and reporting
	• Maintain effective communication with staff
Follow up [11, 17, 22–24, 32]	• Investigate and address all reports of WPV promptly, consistently, and provide appropriate feedback in a debrief after a WPV event
	• Enforce WPV policies
Collaboration [22]	• Collaborate with interprofessional team and management
Support [2, 12, 13, 17, 24]	• Listen to staff and offer post-event support
	• Provide time for staff to formally report WPV
	• Guide staff to additional resources for assistance
	• Message the intolerance of WPV
**Community Interventions**	
Collaboration [6, 11, 13, 24, 26, 32, 34]	• Collaborate with external stakeholders to share data, identify solutions, and implement improvements (e.g. develop campaign on WPV in healthcare, develop global program for tracking and preventing WPV)
	• Promote and sponsor legislation that supports and protects healthcare workers from WPV
	• Collaborate with law enforcement to enforce laws that deter assaults on healthcare workers
	• Work with academia to promote early education for nursing and medical students

*WPV* Workplace Violence

### Limitations

Workplace violence is a complex problem and article retrieval was limited to keyword identification and may have excluded common synonyms for WPV. Accumulation of additional articles via snowball methods is not replicable and may not be complete due to the dynamic topic of WPV. Only quantitative articles published in English were included and may not reflect a complete global understanding of WPV underreporting. Additionally, qualitative studies, editorials, commentaries, and conference abstracts were excluded as authors determined there were substantial qualitative studies to merit a separate analysis.

Content related to the interventions was not initially part of the underreporting systematic review process. Many studies included in the underreporting systematic review contained content about interventions within their study and therefore it was included within the discussion section. A full review of interventions to address underreporting of WPV may be warranted.

## Conclusion

Underreporting is a complex and multi-faceted problem. This systematic review and investigation into the rationale for nurses to not report a WPV event illustrates nurses, management, and organizations contribute to the problem. Without consistent and comprehensive reporting, improvements toward workplace violence solutions within healthcare will continue to be unresolved. Recommendations to address underreporting for each category highlight the importance of clear and actionable reporting procedures, resources, and staff support for reporting.

## Data Availability

The dataset and review materials used during the systematic review are available upon request of the corresponding author.

## Abbreviations

RN: Registered Nurse
WPV: Workplace violence
OSHA: Occupational Safety and Health Administration
PRISMA: Preferred Reporting Items for Systematic Review and Meta-Analysis
CINAHL: Cumulative Index to Nursing and Allied Health Literature
SIGN: Scottish Intercollegiate Guidelines Network
HCW: Healthcare worker
